# Drug Substitution Again

**Published:** 1903-03

**Authors:** 


### Drug Substitution Again.

WE HAVE called attention to the evil of substitution in physicians' prescriptions on several and divers occasions in these columns, but it will do no harm to mention the subject again, because it is a practice that is hard to overcome.

Very recently (January 24, 1903,) *The Medical News*, in an editorial discussion of this subject said:

Commissioner Lederle [of the New York Board of Health] has set in operation a wholesale examination of the stuff that is commonly sold as phenacetin. His physicians and staff have purchased phenacetin powders from 373 drug stores, in Manhattan and Brooklyn. The official report gives the names of all, and includes many well-known drug stores, and department stores. Among these samples, only 58 were found to be pure phenacetin, while the greater number, 315, were adulterated. The chief adulterant was found to be acetanilid, selected undoubtedly because of its cheapness, and its similar effects, in part, to phenacetin.

This would seem almost apocryphal if it were not officially authenticated.

The New York College of Pharmacy has taken action recently on this question in the following terms:

WHEREAS, The substitution of one article when another is called for or of an article of one brand when that of another is ordered, involves an act of deception and an abuse of the confidence of physician or patient, and an act of injustice toward the manufacturer of the article so specified, and,

WHEREAS, The general commission of such acts is destructive of those mutual relations of confidence between manufacturer, pharmacist, physician and patient upon which the highest success of medical practice depends, and,

WHEREAS, Such practises appear to be increasing at the present time, and threatening serious professional and commercial difficulties, therefore, it is

*Resolved*, That the College of Pharmacy of the City of New York publicly condemns all acts of substitution whether in prescription work or in ordinary trade; that it declares such practices to be violations of just dealing, opposed to the principles of professional ethics and subversive of good morals, and it is further

*Resolved*, That we exert our utmost influence both as individuals and as an institution, to discourage such practices and to promote professional and commercial confidence.

It may be noted, as a further point of interest relating to this subject, that Messrs. Farchild Bros. & Foster, of New York, have secured a final decree enjoining Matilda Eberhardt, August F. Eberhardt and John H. Eberhardt,who conduct a drug store at 622 Third Avenue, New York, from selling or dispensing substitutes for Fairchild's essence of pepsine. The injunction carries with it costs for $42.72, together with an extra allowance of $250. This makes it an expensive luxury to indulge in the nefarious practice of substitution, and it is to be hoped that all dishonest druggists will take warning from this most upright decision.

We trust our readers will pay heed to this important matter, and, if need be, interest themselves sufficiently to promote efficient legislation that will prevent a continuance of the evil practice of drug substitution.

